# Contributions of Colonic Short-Chain Fatty Acid Receptors in Energy Homeostasis

**DOI:** 10.3389/fendo.2014.00144

**Published:** 2014-09-02

**Authors:** Atsukazu Kuwahara

**Affiliations:** ^1^Laboratory of Physiology, Graduate School of Integrated Pharmaceutical and Nutritional Sciences, University of Shizuoka, Shizuoka, Japan

**Keywords:** luminal chemosensing, short-chain fatty acid, FFA2, FFA3, energy metabolism, gut microbiota

## Abstract

The gastrointestinal (GI) tract is separated from the body’s internal environment by a single layer of epithelial cells, through which nutrients must pass for their absorption into the bloodstream. Besides food and drink, the GI lumen is also exposed to bioactive chemicals and bacterial products including short-chain fatty acids (SCFAs). Therefore, the GI tract has to monitor the composition of its contents continuously to discriminate between necessary and unnecessary compounds. Recent molecular identification of epithelial membrane receptor proteins has revealed the sensory roles of intestinal epithelial cells in the gut chemosensory system. Malfunctioning of these receptors may be responsible for a variety of metabolic dysfunctions associated with obesity and related disorders. Recent studies suggest that SCFAs produced by microbiota fermentation act as signaling molecules and influence the host’s metabolism; uncovering the sensory mechanisms of such bacterial metabolites would help us understand the interactions between the host and microbiota in host energy homeostasis. In this review, the contribution of colonic SCFA receptors in energy metabolism and our recent findings concerning the possible link between SCFA receptors and host energy homeostasis are discussed.

## Introduction

Part of the gastrointestinal (GI) tract, the intestinal lumen is one-way tube where food materials from the external environment are progressively converted into molecular products. Approximately 100 trillion bacteria, which are termed the gut microbiota, are present in the intestinal lumen, especially in the colon. A single layer of epithelial cells separates this diverse bacterial community from the internal environment. Thus, host–microbiota interactions occur at the mucosal surface of the intestine. The genome of the gut microbiota contains an estimated 150 times as many genes as in the host genome, and continuously produces large amount of various chemicals, including short-chain fatty acids (SCFAs), which can be beneficial or harmful to the host (1). The intestinal lumen is therefore continuously exposed to a multitude of dietary antigens, microorganisms, and bacterial products.

It has become clear that the GI tract responds to a large array of signals in the lumen including nutrient and non-nutrient chemicals. As mentioned above, these responses occur at the level of the mucosa, which contains the epithelial cells. The recent molecular identification of membrane receptor proteins has revealed sensory roles for these epithelial cells in the gut chemosensory system. The failure of functional interactions within the gut chemosensory system between the host and microbiota may cause a spectrum of diseases beyond local GI disorders, such as obesity, diabetes, metabolic syndrome, and various neurological diseases (2). Despite the significant role of the gut microbiota in host health, the elucidation of the molecular mechanistic pathways involved has not been moving forward as expected.

## SCFA Receptors, FFA2 and FFA3 in the Colon

Generally, the main functions of the colon are absorption of water and electrolytes and storage of fecal matter before expulsion. Most studies of the chemosensory system in the GI tract concern the small intestine in relation to nutrient absorption, incretin release, short-term energy control, appetite, satiety, and postprandial glycemic control (3). As a result, for many years chemical sensing in the colon has been considered less important compared with that in the small intestine. However, recent studies suggest that products of microbial metabolisms in the gut act as signaling molecules and influence host energy homeostasis (4).

**Figure 1:**
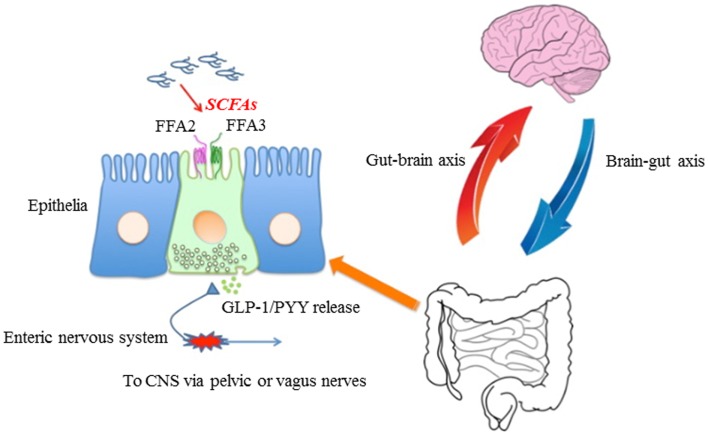
**Proposed model for roles of FFA2/FFA3 that might contribute to host energy homeostasis**. In non-ruminant mammals, short-chain fatty acid (SCFA) is produced by microbiota in the distal small intestine and colon from low-digestible carbohydrates, including resistant starch and soluble oligo- and polysaccharides. These SCFAs are able to bind and activate FFA2 and/or FFA3 located on intestinal epithelia. This activation induces GLP-1 and PYY release into the basolateral side. Released GLP-1 and PYY activate enteric or primary afferent neurons in pelvic and vagal nerves in addition to humoral pathways. These information travel to the CNS, then affect the host metabolic rate to regulate energy homeostasis.

The gut epithelium is composed of different cell types. In the colon, epithelial cells form a sheet consisting of absorptive epithelial, goblet, enteroendocrine, M, and brush (tuft, caveolae) cells (5) (Figure 1). Lamina propria cells and nerve fibers lie close to the epithelial cells but do not directly contact the lumen, and these act together with the cell sheet to monitor luminal contents. Among the epithelial cells, enteroendocrine cells have been proposed to possess intestinal chemosensory function because of their open-type morphology with an apical brush border surface that extends into the gut lumen that comes in contact with chemical compounds (5). Individual enteroendocrine cells are scattered throughout the mucosa, representing ~1% of all epithelial cells in the intestine and comprise a solitary chemosensory system (6). Enteroendocrine cells are subdivided into more than 15–20 different cell types based on their major secretory products and their location along the GI tract (5). A recent study has also reported that certain enteroendocrine cells present in the GI tract express a variety of chemical receptors and gustatory signaling elements such as α-gustducin, α-transducin, and TRPM5 (7).

In 2003, FFA2 (GPR43) and FFA3 (GPR41) were deorphanized as SCFA receptors (8–10). These two receptors share ~40% amino acid sequence similarity and are conserved across several mammalian species. They differ in affinity for SCFAs, tissue distribution, and physiological roles. FFA2 has a similar affinity for acetate, propionate, and butyrate, while FFA3 has a more potent affinity for propionate than butyrate and does not have a high affinity for acetate. Thus, acetate preferentially activates FFA2, propionate mainly activates FFA3, and butyrate equally activates both FFA2 and FFA3. FFA2 and FFA3 also have distinct G proteins coupled in their intracellular signaling cascades; FFA2 couples both pertussis toxin-sensitive (G_i/o_) and toxin-insensitive (G_q_) G proteins, whereas FFA3 couples only to G_i/o_ protein. We hypothesized that FFA2 and FFA3 function as molecular interfaces between the gut microbiota and host colon. Indeed, SCFAs produced by bacterial fermentation in the colon affects local colonic functions; luminal application of propionate and butyrate induces Cl^−^ secretion and muscle contraction in rat distal colons (11, 12). These local effects are not induced by serosal application of SCFAs. Based on these observations, SCFAs are likely to be detected by epithelial cells through specific receptors. We made different antisera for FFA2 and FFA3 using synthesized peptides to test this working hypothesis (13).

Using RT-PCR and western blotting analysis, mRNA and protein for FFA2 were found in the distal ileum and colon in extracts from separated rat mucosa as well as in human colon extracts (13, 14). However, FFA2 mRNA was not detected in submucosal or muscle layers in either species. FFA3 protein and mRNA were detected in human colonic mucosa at higher expression levels than in the submucosal or muscle layer (15). These results suggest the existence of FFA2 and FFA3 in colonic mucosa.

Immunohistochemistry was used to identify the cellular distribution of FFA2 and FFA3 in the colon. FFA2-immunoreactive epithelial cells were found in rat, guinea pig, and human colonic epithelia, with particularly strong expression in PYY and GLP-1-producing enteroendocrine L-cells but not 5-HT containing enterochromaffin (EC) cells (13–16). Immunoreactivity for FFA2 in laboratory animals showed a similar pattern to that of the human colon. FFA2-immunoreactive L-cells in the colon were open-type with bodies extending to the luminal surface. FFA3 was also detected in human colonic open-type L-cells, but it is unclear whether these two receptors are located in the same cells. These morphological characteristics indicate that PYY- and GLP-1-producing L-cells that express FFA2 and FFA3 are chemosensory cells. Activation of these receptors by luminal SCFAs may trigger PYY and/or GLP-1 release. Morphologically, it is still unclear whether FFA2 or FFA3 confined to the apical or basolateral membrane, although our physiological studies indicate that these receptors are located on apical side (11, 12, 16). Further study is needed to clarify the precise distribution pattern of FFA2 and FFA3 in order to understand the physiological function of these receptors.

## Direct Evidence of GLP-1 Release from SCFAs Receptor Expressing L-Cells *In vitro*

The morphological data suggest that gut microbiota-derived SCFAs in the colonic lumen function as stimuli for GLP-1 and PYY release. SCFAs are known to trigger the release of gut hormones, but results have been inconsistent due to differences in the systems used. Enteroendocrine cell culture systems, such as murine STC-1 (17), GLUTag (18), and human NCI-H716 cell lines (19) are often used to study the effects of nutrients on the release gut hormones *in vitro*. The results from single cell cultures, however, are difficult to extrapolate to understand receptor physiological function because intestinal tissue contains different epithelial cell types as well as the enteric nervous system and mucosal immune system, which influence the secretion of gut hormones. In addition, even *in vivo* experiments have inconsistent results; intravenous acetate infusion in innervated and denervated loops in conscious pig did not change the concentration of GLP-1 but did for PYY (20). On the other hand, intravenous and rectal infusion of acetate raises plasma PYY and GLP-1 in hyperinsulinemic human females (21). There are a few possible reasons for such differences: (1) many results of *in vivo* experiments are obtained from an infusion system. This system cannot identify a precise stimulation or secretion site, which is a disadvantage for elucidating the function of chemical receptors in gut hormone secretion. (2) Most cultured cells in *in vitro* cell culture system experiments cannot maintain cell polarity. (3) Many studies cannot directly differentiate whether specific gut hormone-containing enteroendocrine cells are activated to secrete hormones through direct or indirect chemical sensing, particularly if non-enteroendocrine cells also express chemosensory receptors. Indeed, our morphological data suggest that enterocytes also express FFA2 and FFA3.

We used the Ussing chamber system to investigate whether SCFA stimulation induces GLP-1 secretion and to define precise stimulation and secretion sites of FFAs. This preparation maintains the polarity of epithelial cells and contains other cellular elements like intact intestine. In addition, an advantage of this system is that it allows simultaneous measurement of physiological phenomena and hormone release. In muscle-stripped mucosa–submucosal preparations, luminal application of 5 mM propionate induced GLP-1 release into the basolateral side of the rat distal colon (22). Simultaneously, 5 mM propionate induced an increase in short-circuit current, which is a parameter of ion transport in epithelial cells (22). These results show that SCFAs promote GLP-1 secretion through FFAs. It is still unclear, which type of receptor is involved in GLP-1 secretion, since both FFA2 and FFA3 are expressed in enteroendocrine L-cells containing PYY and GLP-1 (13–15). From physiological studies, FFA3 might be involved in this secretion process because acetate, which is the preferable ligand of FFA2, had no effect on local physiological responses including ion transport in the rat distal colon (22). This is further supported by observations of mice lacking FFA2 or FFA3 that had reduced SCFA-triggered GLP-1 secretion *in vitro* and *in vivo* conditions (23). Unfortunately, the molecular pathways underlying the beneficial effects of SCFAs are still largely unknown. Thus, further study is needed to identify molecular pathways of FFA-stimulated GLP-1 secretion.

## Dietary Fiber Supplementation Affects Colonic Enteroendocrine Cell Populations and FFA Expression in the Colon

Besides the direct effects of SCFAs on gut hormone release, some studies have shown a relationship between dietary fiber intake – the substrate for SCFA production by microbiota – and GI hormone release. Indeed, non-digestible and fermentable dietary fibers, as well as SCFAs themselves, have been shown to induce GLP-1 secretion in humans (24) and rodents (25), although the underlying mechanisms are poorly understood. On the other hand, acute dietary fiber intake does not increase endogenous GLP-1 concentration in human subjects (26). To help elucidate these mechanisms, long-term ingestion of fructooligosaccharide (FOS) and its effects on density or expression patterns of FFA2, GLP-1, and 5-HT in the colon were tested using rats. Dietary supplementation with FOS for 4 weeks increased the number of L-cells expressing GLP-1 approximately twofold in the rat proximal colon, but did not affect fecal content or the density of EC cells producing 5-HT (27). These results suggest that luminal SCFAs selectively induce the proliferation of GLP-1-producing cells. This is supported by the observation that long-term ingestion of fermentable dietary fibers increases luminal concentration of SCFAs (28). FFA2 responding to supplementation with 5% FOS approximately doubled in the proximal colon. This suggests that FFA2 plays a key role in GLP-1 production and secretion in addition to FFA3. The microflora environment in the gut must have time to adapt to a new food source before the full effects of fermentation can take effect; production of SCFAs usually requires a few days in animals and humans. As a result, FOS has to be consumed during a long-enough period to allow fermentation to occur and stimulate GLP-1 and PYY production. Therefore, continuous intake of fermentable fiber in the diet is considered important for the expression of GLP-1- and PYY-secreting L-cells in long-term energy homeostasis. As colonic luminal SCFA concentrations are unlikely to be reduced markedly in response to acute food ingestion, it is possible that SCFAs produced by colonic bacterial fermentation provide chronic stimulation to L-cells via FFA2 and FFA3 under physiological condition.

The CNS plays an important role in the maintenance of body weight and energy balance within a narrow range by regulating energy intake and expenditure. A reduction in body weight requires long-term negative energy balance by reducing appetite and food intake or by increasing energy expenditure, or both. GLP-1 and PYY are postulated to be hormonal signals from the gut to the brain to inhibit food intake and appetite control because reduced food intake in part reflects increases in hormone release in conjunction with decreased secretion of ghrelin, which increases food intake through effects on hypothalamic, brainstem, and reward-related circuitry (3). Released GLP-1 is rapidly degraded by DPP-4, which results in a diminished concentration of GLP-1 in the hepatoportal vein (~50%), and an even small amount entering systemic circulation (<10%) (29). Thus, only a small amount of GLP-1 has the chance to reach the CNS. A radiolabeled GLP-1 analog has been shown to cross the blood–brain barrier by simple diffusion in mice, and gut-derived GLP-1 may enter the brain through the area postrema lacking blood–brain barrier (30). Therefore, an alternative model of GLP-1 release from colonic L-cells should be considered. For example, it is possible that continuous release of GLP-1 affects CNS activity to modulate energy homeostasis including appetite and satiety. Alternatively, FFA2/FFA3-activated GLP-1 release from colonic L-cells may activate local gut signaling events to activate the CNS through the pelvic nerve to modulate long-term regulation of appetite and satiety. However, further physiological studies are needed to prove these hypotheses.

GLP-1 is generally believed to be expressed mainly in a limited population of enteroendocrine cells in the ileum and the colon (31). However, recent study suggests that enteroendocrine cells have a much broader potential for expression than is generally believed (32). Furthermore, Li et al. (33) have shown that there are many more L-cells expressing α-gustducin and GLP-1 in the colon than in the small intestine, and that cells expressing α-gustducin increase in distribution from nearly 0% in the small intestine to ~29% in the colon (33), leading the authors to postulate that colonic L-cells might histologically and functionally differ from L-cells in the small intestine. These results suggest that FFA2/FFA3-stimulated GLP-1 secretion from colonic L-cells has a different physiological function than in the small intestine. GLP-1 release is suppressed in obese subjects, so the tonic stimulation of GLP-1 secretion induced by FFAs may chronically affect systemic energy homeostasis (34). Indeed, continuous GLP-1 receptor agonist injection for 14 days has been shown to reduce body weight (35). Therefore, the mode of action of GLP-1/PYY release in the colon stimulated by FFA2 and FFA3 is considered different from the mode of action in the small intestine. Stimulation and release in the colon might be involved in long-term regulation of energy homeostasis, while in the small intestine regulation occurs in the short-term with appetite or feeding control.

## Conclusion

Since obesity and metabolic disorders are associated with changes in gut microbiota, the integral role of the gut microbiota in the regulation of host energy homeostasis is an important issue. SCFAs are primary metabolites of gut bacteria and are present in the colon at high concentrations. The SCFA receptors, FFA2 and FFA3 are located in rodent and human colonic L-cells containing GLP-1 and PYY, whose release are involved in the regulation of host energy homeostasis. Furthermore, luminal application of SCFA induces GLP-1 secretion and long-term ingestion of FOS influences the density of FFA2-expressing L-cells. Therefore, it seems likely that the colonic epithelia communicate with the gut microbiota through products and receptors to maintain long-term host–bacterial interactions. The studies that aimed at elucidating physiological phenomena and underlying mechanisms in the colon discussed in this review are important contributions in the race to develop new therapeutic strategies for obesity and its related disorders in future.

## Conflict of Interest Statement

The author declares that the research was conducted in the absence of any commercial or financial relationships that could be construed as a potential conflict of interest.
